# RETRACTION: Effects of Botulinum Toxin Type A in the Prevention and Treatment of Facial Hypertrophic Scars: A Meta‐Analysis

**DOI:** 10.1111/iwj.70451

**Published:** 2025-03-26

## Abstract

**RETRACTION:** LinJ. and WangX. , “Effects of Botulinum Toxin Type A in the Prevention and Treatment of Facial Hypertrophic Scars: A Meta‐Analysis,” International Wound Journal 21, no. 3 (2024): e14796, 10.1111/iwj.14796.38494191 PMC10944687

The above article, published online on 17 March 2024, in Wiley Online Library (http://onlinelibrary.wiley.com/), has been retracted by agreement between the journal Editor in Chief, Professor Keith Harding; and John Wiley & Sons Ltd. Following an investigation by the publisher, all parties have concluded that this article was accepted solely on the basis of a compromised peer review process. The editors have therefore decided to retract the article. The authors did not respond to our notice regarding the retraction.

**RETRACTION:** J. Lin and X. Wang , “Effects of Botulinum Toxin Type A in the Prevention and Treatment of Facial Hypertrophic Scars: A Meta‐Analysis,” International Wound Journal 21, no. 3 (2024): e14796, 10.1111/iwj.14796.38494191 PMC10944687

The above article, published online on 17 March 2024, in Wiley Online Library (http://onlinelibrary.wiley.com/), has been retracted by agreement between the journal Editor in Chief, Professor Keith Harding; and John Wiley & Sons Ltd. Following an investigation by the publisher, all parties have concluded that this article was accepted solely on the basis of a compromised peer review process. The editors have therefore decided to retract the article. The authors did not respond to our notice regarding the retraction.

